# Carbinoxaminium dipicrate

**DOI:** 10.1107/S1600536813018886

**Published:** 2013-07-17

**Authors:** V. Ramya, Jerry P. Jasinski, James P. Shannon, H. S. Yathirajan, D. K. Ravishankara

**Affiliations:** a Department of Studies in Chemistry, University of Mysore, Manasagangotri, Mysore 570 006, India; b Department of Chemistry, Keene State College, 229 Main Street, Keene, NH 03435-2001, USA; c Sri Mahadeshwara Government First Grade College, Affiliated to University of Mysore, Kollegal 571 440, India

## Abstract

In the dication of the title salt, C_16_H_21_ClN_2_O^2+^·2C_6_H_2_N_3_O_7_^−^ [systematic name: 2-{(4-chloro­phen­yl)[2-(di­methyl­aza­nium­yl)eth­oxy]meth­yl}pyridinium bis­(2,4,6-tri­nitro­phenolate), contains a carbinoxaminium dication and two picrate anions, which are held together through inter­molecular N—H⋯O hydrogen bonds. In the dication, the two aromatic rings form a dihedral angle of 80.1 (1)°. In the two independent picrate anions, the nitro groups are twisted from the benzene plane, the largest dihedral angle in each ion being 42.8 (1) and 81.1 (5)°. In the crystal, in addition to the classical N—H⋯O hydrogen bonds, weak C—H⋯O hydrogen bonds and π–π inter­actions between the aromatic rings of the anions [centroid–centroid distances of 3.5768 (15) and 3.7436 (15) Å] help to establish the packing.

## Related literature

For the pharmacological importance of anti­histamines, see: Wagner (1962 ▶). For the effect of anti­histamines on psychomotor performance, see: Seppala *et al.* (1981 ▶). For related structures, see: Bertolasi *et al.* (1980 ▶); Parvez *et al.* (2001 ▶); Fun *et al.* (2010 ▶); Kaur *et al.* (2013 ▶). For standard bond lengths, see: Allen *et al.* (1987 ▶).
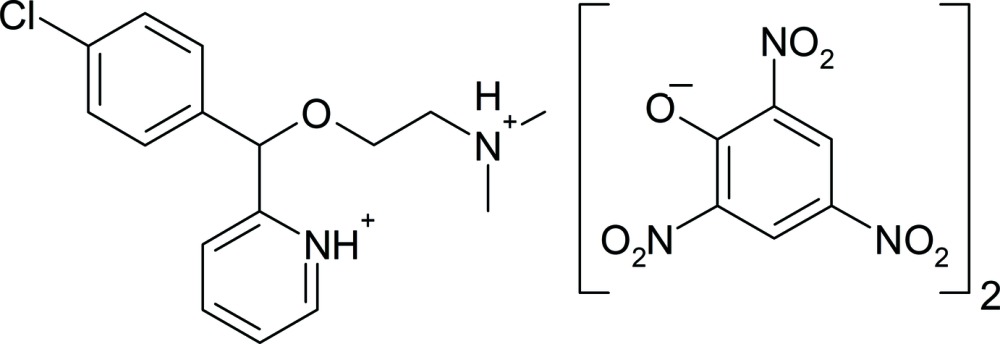


## Experimental

### 

#### Crystal data

C_16_H_21_ClN_2_O^2+^·2C_6_H_2_N_3_O_7_^−^
*M* *_r_* = 749.01
Triclinic, 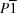

*a* = 8.1719 (6) Å
*b* = 8.5341 (6) Å
*c* = 23.5868 (16) Å
α = 83.771 (6)°
β = 85.484 (6)°
γ = 74.827 (6)°
*V* = 1576.1 (2) Å^3^
*Z* = 2
Cu *K*α radiation
μ = 1.87 mm^−1^
*T* = 173 K
0.24 × 0.16 × 0.12 mm

#### Data collection

Agilent Xcalibur (Eos, Gemini) diffractometer
Absorption correction: multi-scan (*CrysAlis PRO*; Agilent, 2012 ▶) *T*_min_ = 0.871, *T*_max_ = 1.000
9838 measured reflections
6071 independent reflections
4958 reflections with *I* > 2σ(*I*)
*R*_int_ = 0.036

#### Refinement

*R*[*F* ^2^ > 2σ(*F* ^2^)] = 0.050
*wR*(*F* ^2^) = 0.145
*S* = 1.02
6071 reflections
476 parameters
H atoms treated by a mixture of independent and constrained refinement
Δρ_max_ = 0.42 e Å^−3^
Δρ_min_ = −0.38 e Å^−3^

### 

Data collection: *CrysAlis PRO* (Agilent, 2012 ▶); cell refinement: *CrysAlis PRO*; data reduction: *CrysAlis PRO*; program(s) used to solve structure: *SUPERFLIP* (Palatinus & Chapuis, 2007 ▶); program(s) used to refine structure: *SHELXL2012* (Sheldrick, 2008 ▶); molecular graphics: *OLEX2* (Dolomanov *et al.*, 2009 ▶); software used to prepare material for publication: *OLEX2*.

## Supplementary Material

Crystal structure: contains datablock(s) global, I. DOI: 10.1107/S1600536813018886/cv5423sup1.cif

Structure factors: contains datablock(s) I. DOI: 10.1107/S1600536813018886/cv5423Isup2.hkl

Supplementary material file. DOI: 10.1107/S1600536813018886/cv5423Isup3.cml

Additional supplementary materials:  crystallographic information; 3D view; checkCIF report

## Figures and Tables

**Table 1:** Hydrogen-bond geometry (Å, °)

*D*—H⋯*A*	*D*—H	H⋯*A*	*D*⋯*A*	*D*—H⋯*A*
N1—H1⋯O1*B*	0.83 (3)	1.81 (3)	2.628 (2)	167 (3)
N2—H2⋯O1*A*	1.00	1.78	2.737 (3)	159
C1—H1*A*⋯O1*B*	1.00	2.43	3.183 (2)	132
C3—H3⋯O7*A*	0.95	2.53	3.366 (3)	148
C9—H9⋯O2*B* ^i^	0.95	2.38	3.137 (3)	136
C9—H9⋯O7*B*	0.95	2.36	2.956 (3)	120
C11—H11⋯O5*A* ^ii^	0.95	2.49	3.309 (3)	144
C12—H12⋯O1*A*	0.95	2.59	3.430 (3)	148
C14—H14*B*⋯O5*A* ^iii^	0.99	2.46	3.253 (3)	137
C15—H15*B*⋯O4*A* ^iv^	0.98	2.59	3.400 (3)	140
C16—H16*A*⋯O5*B* ^v^	0.98	2.58	3.483 (3)	154

Symmetry codes: (i) 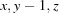; (ii) 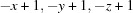; (iii) 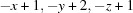; (iv) 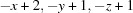; (v) 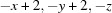.

**Fig. 1.**
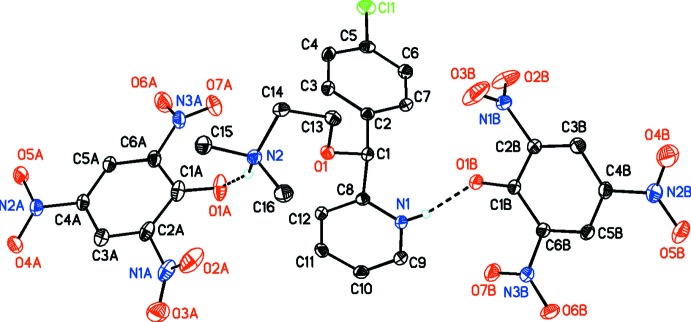
A content of asymmetric unit of (I) showing the atomic labeling and 30% probability displacement ellipsoids. Dashed lines indicate N—H···O hydrogen bonds. C-bound H atoms were omitted for clarity.

## supplementary crystallographic information

### Comment

Carbinoxamine (chemically, 2-[p-chloro-(a)-[2-(dimethylamino)ethoxyl]benzyl] pyridine) is one of the ethanolamine classes of H1 antihistamines and anticholinergic. Antihistamines are drugs used for the treatment of allergic conditions such as urticaria and allergic rhinitis. Due to their well-known sedative side effects most antihistamines are conventionally regarded as detrimental to drivers (Wagner, 1962). However reports of carbinoxamine and other antihistamines being harmless to psychomotor performance and driving skills have been shown (Seppala *et al.*, 1981). A study on carbinoxamine maleate describing the crystallographic structure and chemical relationships of Clistin to other well known antihistaminic drugs and also correlating these chemical aspects with the pharmacological effects produced by this new drug as compared with other antihistaminic agents has been reported by Bertolasi *et al.* (1980). Some number of related structures was reported earlier - orphenadrinium picrate picric acid (Fun *et al.*, 2010); orphenadrinium dihydrogen citrate (Kaur *et al.*, 2013); doxylamine hydrogen succinate (Parvez *et al.*, 2001). In view of the importance of carbinoxamine, this paper reports the crystal structure of the title compound, (I).

The asymmetric unit of (I) (Fig. 1) contains a carbinoxaminium dication and two picrate anions, which are held together through intermolecular N—H···O hydrogen bonds (Table 1). In the dication, the pyridine ring contains a positively charged N atom with quaternary character at the 2 position and a second positive quaternay N atom at the amino group. The two aromatic rings form a dihedral angle of 80.1 (1)°. In picrate anion A, the mean plane of the N1A/C2A/O2A/O3A group is twisted by 42.8 (1)° from the attached benzene ring. In picrate anion B, the mean plane of the N1B/C2B/O2B/O3B group is twisted by 81.1 (5)° from the attached benzene ring. In the crystal, N—H···O hydrogen bonds and weak intermolecular C—H···O interactions between the cation and anion (Table 1) and /p—/p stacking interactions between the benzene rings with the intercentroid distances of 3.5768 (15) and 3.7436 (15) Å contribute to packing stability.

### Experimental

Carbinoxamine succinate (500 mg, 1.18 mmol) and picric acid (270 mg, 1.18 mmol) were dissolved in 20 ml of methanol and stirred for 30 min at 333 K. A yellow precipitate was obtained which was filtered and dried overnight in open air. The obtained compound was then recrystallized from a 1:1 solution of benzene and dimethylsulphoxide by the slow evaporation method. (m.p.: 423–438 K).

### Refinement

Amino atom H1 was located on a difference map and isotropically refined. All the rest H atoms were geometrically positioned (C—H 0.95–1.00 Å; N—H 1.00 Å), and then refined as riding, with *U*_iso_ =1.2 – 1.5 *U*_eq_ of the parent atom.

### Crystal data

**Table:**

C_16_H_21_ClN_2_O^2+^·2C_6_H_2_N_3_O_7_^−^	*Z* = 2
*M**_r_* = 749.01	*F*(000) = 772
Triclinic, *P*1	*D*_x_ = 1.578 Mg m^−^^3^
*a* = 8.1719 (6) Å	Cu *K*α radiation, λ = 1.54184 Å
*b* = 8.5341 (6) Å	Cell parameters from 3773 reflections
*c* = 23.5868 (16) Å	θ = 3.8–72.4°
α = 83.771 (6)°	µ = 1.87 mm^−^^1^
β = 85.484 (6)°	*T* = 173 K
γ = 74.827 (6)°	Prism, yellow
*V* = 1576.1 (2) Å^3^	0.24 × 0.16 × 0.12 mm

### Data collection

**Table:**

Agilent Xcalibur (Eos, Gemini) diffractometer	6071 independent reflections
Radiation source: Enhance (Cu) X-ray Source	4958 reflections with *I* > 2σ(*I*)
Detector resolution: 16.0416 pixels mm^-1^	*R*_int_ = 0.036
ω scans	θ_max_ = 72.5°, θ_min_ = 3.8°
Absorption correction: multi-scan (*CrysAlis PRO*; Agilent, 2012)	*h* = −9→10
*T*_min_ = 0.871, *T*_max_ = 1.000	*k* = −6→10
9838 measured reflections	*l* = −26→28

### Refinement

**Table:**

Refinement on *F*^2^	Hydrogen site location: inferred from neighbouring sites
Least-squares matrix: full	H atoms treated by a mixture of independent and constrained refinement
*R*[*F*^2^ > 2σ(*F*^2^)] = 0.050	*w* = 1/[σ^2^(*F*_o_^2^) + (0.0778*P*)^2^ + 0.3489*P*] where *P* = (*F*_o_^2^ + 2*F*_c_^2^)/3
*wR*(*F*^2^) = 0.145	(Δ/σ)_max_ < 0.001
*S* = 1.02	Δρ_max_ = 0.42 e Å^−^^3^
6071 reflections	Δρ_min_ = −0.38 e Å^−^^3^
476 parameters	Extinction correction: *SHELXL*, Fc^*^=kFc[1+0.001xFc^2^λ^3^/sin(2θ)]^-1/4^
0 restraints	Extinction coefficient: 0.0006 (3)

### Special details

**Table:**

Geometry. All esds (except the esd in the dihedral angle between two l.s. planes) are estimated using the full covariance matrix. The cell esds are taken into account individually in the estimation of esds in distances, angles and torsion angles; correlations between esds in cell parameters are only used when they are defined by crystal symmetry. An approximate (isotropic) treatment of cell esds is used for estimating esds involving l.s. planes.

### Fractional atomic coordinates and isotropic or equivalent isotropic displacement parameters (Å^2^)

**Table:**

	*x*	*y*	*z*	*U*_iso_*/*U*_eq_	
Cl1	−0.11261 (7)	1.50201 (7)	0.24701 (3)	0.04328 (18)	
O1	0.66697 (18)	0.99915 (17)	0.25648 (6)	0.0256 (3)	
N1	0.5260 (2)	0.8100 (2)	0.15238 (8)	0.0259 (4)	
H1	0.555 (4)	0.868 (4)	0.1249 (13)	0.045 (8)*	
N2	0.9121 (2)	0.9449 (2)	0.33517 (7)	0.0270 (4)	
H2	0.8313	0.8739	0.3411	0.032*	
C1	0.5639 (2)	1.0201 (2)	0.20923 (8)	0.0229 (4)	
H1A	0.6251	1.0540	0.1735	0.027*	
C2	0.3938 (3)	1.1451 (2)	0.21871 (9)	0.0232 (4)	
C3	0.3387 (3)	1.1925 (3)	0.27286 (9)	0.0273 (4)	
H3	0.4076	1.1482	0.3043	0.033*	
C4	0.1838 (3)	1.3039 (3)	0.28151 (9)	0.0296 (5)	
H4	0.1471	1.3382	0.3184	0.036*	
C5	0.0832 (3)	1.3645 (3)	0.23545 (10)	0.0288 (5)	
C6	0.1341 (3)	1.3170 (3)	0.18120 (10)	0.0304 (5)	
H6	0.0630	1.3585	0.1501	0.037*	
C7	0.2907 (3)	1.2077 (3)	0.17307 (9)	0.0274 (4)	
H7	0.3281	1.1752	0.1360	0.033*	
C8	0.5295 (2)	0.8557 (2)	0.20483 (9)	0.0232 (4)	
C9	0.4844 (3)	0.6731 (3)	0.14378 (10)	0.0311 (5)	
H9	0.4842	0.6444	0.1060	0.037*	
C10	0.4421 (3)	0.5740 (3)	0.18928 (10)	0.0314 (5)	
H10	0.4096	0.4783	0.1833	0.038*	
C11	0.4475 (3)	0.6162 (3)	0.24404 (9)	0.0298 (5)	
H11	0.4208	0.5479	0.2761	0.036*	
C12	0.4921 (3)	0.7583 (2)	0.25224 (9)	0.0260 (4)	
H12	0.4968	0.7879	0.2896	0.031*	
C13	0.7505 (3)	1.1265 (3)	0.25669 (9)	0.0278 (4)	
H13A	0.6705	1.2343	0.2480	0.033*	
H13B	0.8467	1.1132	0.2278	0.033*	
C14	0.8134 (3)	1.1129 (3)	0.31594 (9)	0.0285 (4)	
H14A	0.8860	1.1890	0.3166	0.034*	
H14B	0.7149	1.1464	0.3432	0.034*	
C15	0.9831 (4)	0.9471 (3)	0.39156 (11)	0.0432 (6)	
H15A	1.0624	1.0166	0.3871	0.065*	
H15B	1.0429	0.8360	0.4054	0.065*	
H15C	0.8903	0.9902	0.4191	0.065*	
C16	1.0494 (3)	0.8722 (3)	0.29313 (11)	0.0375 (5)	
H16A	0.9990	0.8586	0.2582	0.056*	
H16B	1.1142	0.7656	0.3095	0.056*	
H16C	1.1252	0.9444	0.2840	0.056*	
O1A	0.6767 (2)	0.7783 (3)	0.37604 (7)	0.0510 (5)	
O2A	0.9906 (3)	0.5583 (3)	0.38556 (9)	0.0628 (6)	
O3A	0.9285 (3)	0.3338 (3)	0.41788 (10)	0.0648 (6)	
O4A	0.6720 (2)	0.3773 (2)	0.61350 (7)	0.0384 (4)	
O5A	0.4787 (3)	0.5957 (2)	0.63041 (7)	0.0499 (5)	
O6A	0.2565 (3)	0.9728 (3)	0.47738 (9)	0.0603 (6)	
O7A	0.4233 (4)	1.0273 (3)	0.40692 (9)	0.0810 (8)	
N1A	0.9039 (3)	0.4822 (3)	0.41567 (9)	0.0450 (5)	
N2A	0.5868 (2)	0.5149 (2)	0.59866 (8)	0.0323 (4)	
N3A	0.3898 (3)	0.9401 (3)	0.44864 (9)	0.0467 (6)	
C1A	0.6573 (3)	0.7231 (3)	0.42626 (9)	0.0360 (5)	
C2A	0.7648 (3)	0.5717 (3)	0.45134 (10)	0.0348 (5)	
C3A	0.7429 (3)	0.5018 (3)	0.50562 (9)	0.0328 (5)	
H3A	0.8156	0.4001	0.5186	0.039*	
C4A	0.6116 (3)	0.5837 (3)	0.54113 (9)	0.0288 (4)	
C5A	0.4993 (3)	0.7266 (3)	0.52136 (9)	0.0306 (5)	
H5A	0.4078	0.7790	0.5457	0.037*	
C6A	0.5199 (3)	0.7930 (3)	0.46644 (9)	0.0333 (5)	
O1B	0.5906 (2)	1.03134 (19)	0.07357 (7)	0.0357 (4)	
O2B	0.5038 (3)	1.3996 (3)	0.06288 (11)	0.0659 (7)	
O3B	0.7430 (4)	1.3352 (4)	0.09961 (11)	0.0893 (10)	
O4B	1.0327 (4)	1.3210 (3)	−0.11434 (11)	0.0780 (8)	
O5B	1.0417 (3)	1.0971 (3)	−0.14993 (8)	0.0573 (6)	
O6B	0.8368 (2)	0.6875 (2)	−0.03911 (8)	0.0453 (5)	
O7B	0.6563 (3)	0.7393 (2)	0.03210 (8)	0.0549 (6)	
N1B	0.6504 (3)	1.3270 (2)	0.06334 (8)	0.0350 (4)	
N2B	0.9919 (3)	1.1925 (3)	−0.11323 (9)	0.0426 (5)	
N3B	0.7522 (3)	0.7805 (2)	−0.00549 (8)	0.0336 (4)	
C1B	0.6833 (3)	1.0589 (3)	0.03035 (9)	0.0270 (4)	
C2B	0.7212 (3)	1.2152 (3)	0.01923 (9)	0.0286 (4)	
C3B	0.8166 (3)	1.2631 (3)	−0.02558 (9)	0.0308 (5)	
H3B	0.8360	1.3689	−0.0299	0.037*	
C4B	0.8853 (3)	1.1496 (3)	−0.06533 (9)	0.0304 (5)	
C5B	0.8615 (3)	0.9943 (3)	−0.05819 (9)	0.0291 (5)	
H5B	0.9116	0.9185	−0.0852	0.035*	
C6B	0.7647 (3)	0.9490 (3)	−0.01178 (9)	0.0272 (4)	

### Atomic displacement parameters (Å^2^)

**Table:**

	*U*^11^	*U*^22^	*U*^33^	*U*^12^	*U*^13^	*U*^23^
Cl1	0.0298 (3)	0.0375 (3)	0.0553 (4)	0.0028 (2)	0.0052 (2)	−0.0063 (3)
O1	0.0273 (7)	0.0240 (7)	0.0269 (7)	−0.0089 (6)	−0.0051 (6)	−0.0002 (6)
N1	0.0313 (9)	0.0245 (9)	0.0216 (9)	−0.0076 (7)	0.0021 (7)	−0.0014 (7)
N2	0.0272 (9)	0.0278 (9)	0.0258 (9)	−0.0056 (7)	−0.0026 (7)	−0.0032 (7)
C1	0.0253 (10)	0.0231 (9)	0.0201 (9)	−0.0060 (8)	−0.0006 (7)	−0.0019 (7)
C2	0.0253 (10)	0.0203 (9)	0.0255 (10)	−0.0085 (8)	0.0002 (8)	−0.0027 (8)
C3	0.0280 (10)	0.0300 (11)	0.0247 (10)	−0.0091 (8)	−0.0018 (8)	−0.0024 (8)
C4	0.0314 (11)	0.0334 (11)	0.0249 (10)	−0.0100 (9)	0.0055 (8)	−0.0070 (9)
C5	0.0229 (10)	0.0230 (10)	0.0391 (12)	−0.0040 (8)	0.0043 (8)	−0.0051 (9)
C6	0.0291 (11)	0.0296 (11)	0.0327 (12)	−0.0068 (9)	−0.0056 (9)	−0.0019 (9)
C7	0.0318 (11)	0.0252 (10)	0.0256 (10)	−0.0066 (8)	−0.0012 (8)	−0.0055 (8)
C8	0.0196 (9)	0.0232 (9)	0.0256 (10)	−0.0032 (7)	0.0001 (7)	−0.0038 (8)
C9	0.0380 (12)	0.0285 (11)	0.0268 (11)	−0.0070 (9)	−0.0009 (9)	−0.0066 (9)
C10	0.0372 (12)	0.0233 (10)	0.0355 (12)	−0.0101 (9)	−0.0002 (9)	−0.0058 (9)
C11	0.0330 (11)	0.0265 (10)	0.0278 (11)	−0.0069 (9)	0.0017 (8)	0.0030 (8)
C12	0.0290 (10)	0.0260 (10)	0.0218 (10)	−0.0047 (8)	−0.0005 (8)	−0.0033 (8)
C13	0.0255 (10)	0.0251 (10)	0.0344 (11)	−0.0092 (8)	−0.0035 (8)	−0.0010 (8)
C14	0.0275 (10)	0.0245 (10)	0.0341 (11)	−0.0058 (8)	−0.0028 (8)	−0.0067 (9)
C15	0.0522 (15)	0.0412 (14)	0.0344 (13)	−0.0036 (11)	−0.0166 (11)	−0.0057 (11)
C16	0.0297 (12)	0.0378 (13)	0.0400 (13)	−0.0007 (10)	0.0022 (10)	−0.0051 (10)
O1A	0.0498 (11)	0.0875 (15)	0.0252 (9)	−0.0400 (11)	−0.0032 (7)	0.0092 (9)
O2A	0.0600 (13)	0.0879 (16)	0.0548 (12)	−0.0453 (12)	0.0332 (10)	−0.0307 (12)
O3A	0.0659 (14)	0.0592 (14)	0.0602 (14)	−0.0059 (11)	0.0231 (11)	−0.0100 (11)
O4A	0.0377 (9)	0.0383 (9)	0.0337 (9)	−0.0026 (7)	−0.0031 (7)	0.0050 (7)
O5A	0.0605 (12)	0.0493 (11)	0.0245 (9)	0.0068 (9)	0.0119 (8)	0.0020 (8)
O6A	0.0566 (13)	0.0600 (13)	0.0517 (12)	0.0047 (10)	−0.0095 (10)	0.0065 (10)
O7A	0.146 (3)	0.0492 (13)	0.0382 (12)	−0.0148 (15)	−0.0048 (13)	0.0148 (10)
N1A	0.0419 (12)	0.0678 (16)	0.0320 (11)	−0.0237 (11)	0.0082 (9)	−0.0171 (10)
N2A	0.0321 (10)	0.0389 (11)	0.0243 (9)	−0.0074 (8)	−0.0011 (7)	0.0000 (8)
N3A	0.0757 (17)	0.0401 (12)	0.0274 (11)	−0.0182 (11)	−0.0177 (11)	0.0027 (9)
C1A	0.0403 (13)	0.0547 (15)	0.0233 (11)	−0.0318 (11)	−0.0018 (9)	−0.0007 (10)
C2A	0.0324 (12)	0.0527 (14)	0.0254 (11)	−0.0205 (11)	0.0045 (9)	−0.0106 (10)
C3A	0.0312 (12)	0.0421 (13)	0.0275 (11)	−0.0132 (10)	−0.0007 (9)	−0.0053 (9)
C4A	0.0324 (11)	0.0355 (11)	0.0202 (10)	−0.0123 (9)	−0.0005 (8)	−0.0011 (8)
C5A	0.0348 (11)	0.0357 (12)	0.0230 (10)	−0.0117 (9)	−0.0016 (8)	−0.0030 (9)
C6A	0.0433 (13)	0.0361 (12)	0.0254 (11)	−0.0183 (10)	−0.0067 (9)	0.0001 (9)
O1B	0.0498 (10)	0.0294 (8)	0.0262 (8)	−0.0103 (7)	0.0101 (7)	−0.0037 (6)
O2B	0.0407 (11)	0.0705 (15)	0.0901 (17)	−0.0047 (10)	0.0063 (11)	−0.0533 (13)
O3B	0.0874 (18)	0.102 (2)	0.0667 (16)	0.0240 (15)	−0.0328 (14)	−0.0554 (15)
O4B	0.111 (2)	0.0569 (14)	0.0702 (15)	−0.0414 (14)	0.0510 (15)	−0.0147 (12)
O5B	0.0684 (14)	0.0647 (13)	0.0369 (10)	−0.0175 (11)	0.0225 (9)	−0.0134 (9)
O6B	0.0565 (11)	0.0355 (9)	0.0443 (10)	−0.0106 (8)	0.0101 (8)	−0.0175 (8)
O7B	0.0897 (16)	0.0415 (11)	0.0377 (10)	−0.0289 (11)	0.0213 (10)	−0.0097 (8)
N1B	0.0470 (12)	0.0279 (10)	0.0313 (10)	−0.0121 (9)	0.0048 (9)	−0.0072 (8)
N2B	0.0453 (12)	0.0437 (12)	0.0347 (11)	−0.0090 (10)	0.0115 (9)	−0.0019 (9)
N3B	0.0447 (11)	0.0316 (10)	0.0250 (9)	−0.0092 (8)	−0.0022 (8)	−0.0055 (8)
C1B	0.0310 (11)	0.0280 (10)	0.0209 (10)	−0.0050 (8)	−0.0020 (8)	−0.0025 (8)
C2B	0.0313 (11)	0.0278 (10)	0.0246 (10)	−0.0039 (8)	0.0010 (8)	−0.0041 (8)
C3B	0.0312 (11)	0.0294 (11)	0.0305 (11)	−0.0067 (9)	0.0002 (9)	−0.0012 (9)
C4B	0.0308 (11)	0.0349 (12)	0.0230 (10)	−0.0062 (9)	0.0021 (8)	0.0001 (9)
C5B	0.0308 (11)	0.0327 (11)	0.0213 (10)	−0.0022 (9)	−0.0022 (8)	−0.0059 (8)
C6B	0.0308 (11)	0.0273 (10)	0.0228 (10)	−0.0051 (8)	−0.0032 (8)	−0.0031 (8)

### Geometric parameters (Å, º)

**Table:**

Cl1—C5	1.741 (2)	C16—H16A	0.9800
O1—C1	1.418 (2)	C16—H16B	0.9800
O1—C13	1.428 (2)	C16—H16C	0.9800
N1—H1	0.83 (3)	O1A—C1A	1.240 (3)
N1—C8	1.341 (3)	O2A—N1A	1.220 (3)
N1—C9	1.338 (3)	O3A—N1A	1.226 (3)
N2—H2	1.0000	O4A—N2A	1.227 (3)
N2—C14	1.491 (3)	O5A—N2A	1.227 (3)
N2—C15	1.496 (3)	O6A—N3A	1.220 (3)
N2—C16	1.490 (3)	O7A—N3A	1.226 (3)
C1—H1A	1.0000	N1A—C2A	1.455 (3)
C1—C2	1.532 (3)	N2A—C4A	1.440 (3)
C1—C8	1.517 (3)	N3A—C6A	1.463 (3)
C2—C3	1.387 (3)	C1A—C2A	1.454 (4)
C2—C7	1.392 (3)	C1A—C6A	1.456 (3)
C3—H3	0.9500	C2A—C3A	1.371 (3)
C3—C4	1.386 (3)	C3A—H3A	0.9500
C4—H4	0.9500	C3A—C4A	1.391 (3)
C4—C5	1.385 (3)	C4A—C5A	1.380 (3)
C5—C6	1.384 (3)	C5A—H5A	0.9500
C6—H6	0.9500	C5A—C6A	1.372 (3)
C6—C7	1.386 (3)	O1B—C1B	1.260 (3)
C7—H7	0.9500	O2B—N1B	1.196 (3)
C8—C12	1.382 (3)	O3B—N1B	1.205 (3)
C9—H9	0.9500	O4B—N2B	1.224 (3)
C9—C10	1.373 (3)	O5B—N2B	1.225 (3)
C10—H10	0.9500	O6B—N3B	1.225 (2)
C10—C11	1.385 (3)	O7B—N3B	1.221 (3)
C11—H11	0.9500	N1B—C2B	1.469 (3)
C11—C12	1.391 (3)	N2B—C4B	1.446 (3)
C12—H12	0.9500	N3B—C6B	1.459 (3)
C13—H13A	0.9900	C1B—C2B	1.439 (3)
C13—H13B	0.9900	C1B—C6B	1.443 (3)
C13—C14	1.509 (3)	C2B—C3B	1.355 (3)
C14—H14A	0.9900	C3B—H3B	0.9500
C14—H14B	0.9900	C3B—C4B	1.401 (3)
C15—H15A	0.9800	C4B—C5B	1.380 (3)
C15—H15B	0.9800	C5B—H5B	0.9500
C15—H15C	0.9800	C5B—C6B	1.380 (3)
			
C1—O1—C13	112.85 (15)	H15A—C15—H15B	109.5
C8—N1—H1	118 (2)	H15A—C15—H15C	109.5
C9—N1—H1	120 (2)	H15B—C15—H15C	109.5
C9—N1—C8	122.27 (19)	N2—C16—H16A	109.5
C14—N2—H2	107.7	N2—C16—H16B	109.5
C14—N2—C15	109.09 (17)	N2—C16—H16C	109.5
C15—N2—H2	107.7	H16A—C16—H16B	109.5
C16—N2—H2	107.7	H16A—C16—H16C	109.5
C16—N2—C14	113.72 (17)	H16B—C16—H16C	109.5
C16—N2—C15	110.69 (19)	O2A—N1A—O3A	123.5 (2)
O1—C1—H1A	110.2	O2A—N1A—C2A	118.5 (2)
O1—C1—C2	111.44 (15)	O3A—N1A—C2A	118.0 (2)
O1—C1—C8	106.13 (16)	O4A—N2A—C4A	118.84 (19)
C2—C1—H1A	110.2	O5A—N2A—O4A	122.87 (19)
C8—C1—H1A	110.2	O5A—N2A—C4A	118.27 (19)
C8—C1—C2	108.51 (16)	O6A—N3A—O7A	123.5 (3)
C3—C2—C1	120.58 (18)	O6A—N3A—C6A	118.3 (2)
C3—C2—C7	119.51 (19)	O7A—N3A—C6A	118.1 (3)
C7—C2—C1	119.87 (17)	O1A—C1A—C2A	123.5 (2)
C2—C3—H3	119.7	O1A—C1A—C6A	125.4 (3)
C4—C3—C2	120.5 (2)	C2A—C1A—C6A	111.06 (19)
C4—C3—H3	119.7	C1A—C2A—N1A	117.9 (2)
C3—C4—H4	120.5	C3A—C2A—N1A	116.6 (2)
C5—C4—C3	118.96 (19)	C3A—C2A—C1A	125.5 (2)
C5—C4—H4	120.5	C2A—C3A—H3A	120.8
C4—C5—Cl1	118.57 (17)	C2A—C3A—C4A	118.3 (2)
C6—C5—Cl1	119.81 (18)	C4A—C3A—H3A	120.8
C6—C5—C4	121.6 (2)	C3A—C4A—N2A	119.7 (2)
C5—C6—H6	120.6	C5A—C4A—N2A	119.2 (2)
C5—C6—C7	118.7 (2)	C5A—C4A—C3A	121.1 (2)
C7—C6—H6	120.6	C4A—C5A—H5A	120.0
C2—C7—H7	119.7	C6A—C5A—C4A	119.9 (2)
C6—C7—C2	120.67 (19)	C6A—C5A—H5A	120.0
C6—C7—H7	119.7	C1A—C6A—N3A	120.1 (2)
N1—C8—C1	117.61 (18)	C5A—C6A—N3A	115.8 (2)
N1—C8—C12	119.82 (19)	C5A—C6A—C1A	124.0 (2)
C12—C8—C1	122.46 (18)	O2B—N1B—O3B	123.5 (2)
N1—C9—H9	119.8	O2B—N1B—C2B	118.9 (2)
N1—C9—C10	120.3 (2)	O3B—N1B—C2B	117.5 (2)
C10—C9—H9	119.8	O4B—N2B—O5B	122.8 (2)
C9—C10—H10	120.6	O4B—N2B—C4B	118.2 (2)
C9—C10—C11	118.8 (2)	O5B—N2B—C4B	119.0 (2)
C11—C10—H10	120.6	O6B—N3B—C6B	117.99 (19)
C10—C11—H11	120.0	O7B—N3B—O6B	123.0 (2)
C10—C11—C12	120.1 (2)	O7B—N3B—C6B	118.96 (19)
C12—C11—H11	120.0	O1B—C1B—C2B	119.94 (19)
C8—C12—C11	118.64 (19)	O1B—C1B—C6B	127.9 (2)
C8—C12—H12	120.7	C2B—C1B—C6B	112.14 (19)
C11—C12—H12	120.7	C1B—C2B—N1B	113.88 (18)
O1—C13—H13A	110.5	C3B—C2B—N1B	119.5 (2)
O1—C13—H13B	110.5	C3B—C2B—C1B	126.6 (2)
O1—C13—C14	106.08 (17)	C2B—C3B—H3B	121.5
H13A—C13—H13B	108.7	C2B—C3B—C4B	116.9 (2)
C14—C13—H13A	110.5	C4B—C3B—H3B	121.5
C14—C13—H13B	110.5	C3B—C4B—N2B	119.4 (2)
N2—C14—C13	113.08 (16)	C5B—C4B—N2B	119.0 (2)
N2—C14—H14A	109.0	C5B—C4B—C3B	121.5 (2)
N2—C14—H14B	109.0	C4B—C5B—H5B	120.0
C13—C14—H14A	109.0	C6B—C5B—C4B	120.00 (19)
C13—C14—H14B	109.0	C6B—C5B—H5B	120.0
H14A—C14—H14B	107.8	C1B—C6B—N3B	120.33 (19)
N2—C15—H15A	109.5	C5B—C6B—N3B	116.93 (18)
N2—C15—H15B	109.5	C5B—C6B—C1B	122.7 (2)
N2—C15—H15C	109.5		
			
Cl1—C5—C6—C7	179.84 (16)	O6A—N3A—C6A—C1A	162.2 (2)
O1—C1—C2—C3	−15.7 (3)	O6A—N3A—C6A—C5A	−15.9 (3)
O1—C1—C2—C7	166.52 (17)	O7A—N3A—C6A—C1A	−20.6 (3)
O1—C1—C8—N1	−142.31 (17)	O7A—N3A—C6A—C5A	161.2 (2)
O1—C1—C8—C12	41.7 (2)	N1A—C2A—C3A—C4A	179.83 (19)
O1—C13—C14—N2	50.9 (2)	N2A—C4A—C5A—C6A	−179.3 (2)
N1—C8—C12—C11	−1.5 (3)	C1A—C2A—C3A—C4A	−2.6 (3)
N1—C9—C10—C11	−1.7 (3)	C2A—C1A—C6A—N3A	−175.87 (19)
C1—O1—C13—C14	164.84 (16)	C2A—C1A—C6A—C5A	2.1 (3)
C1—C2—C3—C4	−179.11 (19)	C2A—C3A—C4A—N2A	−179.0 (2)
C1—C2—C7—C6	177.94 (18)	C2A—C3A—C4A—C5A	3.9 (3)
C1—C8—C12—C11	174.37 (19)	C3A—C4A—C5A—C6A	−2.3 (3)
C2—C1—C8—N1	97.8 (2)	C4A—C5A—C6A—N3A	177.1 (2)
C2—C1—C8—C12	−78.2 (2)	C4A—C5A—C6A—C1A	−1.0 (3)
C2—C3—C4—C5	1.4 (3)	C6A—C1A—C2A—N1A	177.21 (19)
C3—C2—C7—C6	0.2 (3)	C6A—C1A—C2A—C3A	−0.3 (3)
C3—C4—C5—Cl1	179.02 (16)	O1B—C1B—C2B—N1B	2.9 (3)
C3—C4—C5—C6	−0.3 (3)	O1B—C1B—C2B—C3B	−179.1 (2)
C4—C5—C6—C7	−0.9 (3)	O1B—C1B—C6B—N3B	−3.4 (4)
C5—C6—C7—C2	0.9 (3)	O1B—C1B—C6B—C5B	178.8 (2)
C7—C2—C3—C4	−1.3 (3)	O2B—N1B—C2B—C1B	−79.9 (3)
C8—N1—C9—C10	0.5 (3)	O2B—N1B—C2B—C3B	102.0 (3)
C8—C1—C2—C3	100.8 (2)	O3B—N1B—C2B—C1B	97.2 (3)
C8—C1—C2—C7	−77.0 (2)	O3B—N1B—C2B—C3B	−80.9 (3)
C9—N1—C8—C1	−174.95 (19)	O4B—N2B—C4B—C3B	7.3 (4)
C9—N1—C8—C12	1.2 (3)	O4B—N2B—C4B—C5B	−169.1 (3)
C9—C10—C11—C12	1.2 (3)	O5B—N2B—C4B—C3B	−174.9 (2)
C10—C11—C12—C8	0.4 (3)	O5B—N2B—C4B—C5B	8.6 (4)
C13—O1—C1—C2	−79.0 (2)	O6B—N3B—C6B—C1B	−172.9 (2)
C13—O1—C1—C8	163.01 (16)	O6B—N3B—C6B—C5B	5.0 (3)
C15—N2—C14—C13	174.50 (19)	O7B—N3B—C6B—C1B	8.3 (3)
C16—N2—C14—C13	50.4 (2)	O7B—N3B—C6B—C5B	−173.8 (2)
O1A—C1A—C2A—N1A	1.1 (3)	N1B—C2B—C3B—C4B	178.0 (2)
O1A—C1A—C2A—C3A	−176.5 (2)	N2B—C4B—C5B—C6B	177.9 (2)
O1A—C1A—C6A—N3A	0.2 (3)	C1B—C2B—C3B—C4B	0.1 (4)
O1A—C1A—C6A—C5A	178.2 (2)	C2B—C1B—C6B—N3B	175.34 (19)
O2A—N1A—C2A—C1A	43.5 (3)	C2B—C1B—C6B—C5B	−2.5 (3)
O2A—N1A—C2A—C3A	−138.8 (3)	C2B—C3B—C4B—N2B	−178.4 (2)
O3A—N1A—C2A—C1A	−137.1 (3)	C2B—C3B—C4B—C5B	−2.0 (3)
O3A—N1A—C2A—C3A	40.7 (3)	C3B—C4B—C5B—C6B	1.6 (3)
O4A—N2A—C4A—C3A	−7.0 (3)	C4B—C5B—C6B—N3B	−177.0 (2)
O4A—N2A—C4A—C5A	170.1 (2)	C4B—C5B—C6B—C1B	0.9 (3)
O5A—N2A—C4A—C3A	174.4 (2)	C6B—C1B—C2B—N1B	−175.96 (18)
O5A—N2A—C4A—C5A	−8.5 (3)	C6B—C1B—C2B—C3B	2.0 (3)

### Hydrogen-bond geometry (Å, º)

**Table:**

*D*—H···*A*	*D*—H	H···*A*	*D*···*A*	*D*—H···*A*
N1—H1···O1*B*	0.83 (3)	1.81 (3)	2.628 (2)	167 (3)
N2—H2···O1*A*	1.00	1.78	2.737 (3)	159
C1—H1*A*···O1*B*	1.00	2.43	3.183 (2)	132
C3—H3···O7*A*	0.95	2.53	3.366 (3)	148
C9—H9···O2*B*^i^	0.95	2.38	3.137 (3)	136
C9—H9···O7*B*	0.95	2.36	2.956 (3)	120
C11—H11···O5*A*^ii^	0.95	2.49	3.309 (3)	144
C12—H12···O1*A*	0.95	2.59	3.430 (3)	148
C14—H14*B*···O5*A*^iii^	0.99	2.46	3.253 (3)	137
C15—H15*B*···O4*A*^iv^	0.98	2.59	3.400 (3)	140
C16—H16*A*···O5*B*^v^	0.98	2.58	3.483 (3)	154

Symmetry codes: (i) *x*, *y*−1, *z*; (ii) −*x*+1, −*y*+1, −*z*+1; (iii) −*x*+1, −*y*+2, −*z*+1; (iv) −*x*+2, −*y*+1, −*z*+1; (v) −*x*+2, −*y*+2, −*z*.

## References

[bb1] Agilent (2012). *CrysAlis PRO* Agilent Technologies, Yarnton, England.

[bb2] Allen, F. H., Kennard, O., Watson, D. G., Brammer, L., Orpen, A. G. & Taylor, R. (1987). *J. Chem. Soc. Perkin Trans. 2*, pp. S1–19.

[bb3] Bertolasi, V., Borea, P. A., Gilli, G. & Sacerdoti, M. (1980). *Acta Cryst.* B**36**, 2287–2291.

[bb4] Dolomanov, O. V., Bourhis, L. J., Gildea, R. J., Howard, J. A. K. & Puschmann, H. (2009). *J. Appl. Cryst.* **42**, 339–341.

[bb5] Fun, H.-K., Hemamalini, M., Siddaraju, B. P., Yathirajan, H. S. & Narayana, B. (2010). *Acta Cryst.* E**66**, o682–o683. 10.1107/S1600536810006379 PMC2983644 21580426

[bb6] Kaur, M., Jasinski, J. P., Keeley, A. C., Yathirajan, H. S. & Siddaraju, B. P. (2013). *Acta Cryst.* E**69**, o248. 10.1107/S1600536813001207 PMC3569781 23424527

[bb7] Palatinus, L. & Chapuis, G. (2007). *J. Appl. Cryst.* **40**, 786–790.

[bb8] Parvez, M., Dalrymple, S. & Cote, A. (2001). *Acta Cryst.* E**57**, o163–o165.

[bb9] Seppala, T., Nuotto, E. & Korttila, K. (1981). *Br. J. Clin. Pharmacol.* **12**, 179–188. 10.1111/j.1365-2125.1981.tb01198.x PMC1401857 6118170

[bb10] Sheldrick, G. M. (2008). *Acta Cryst.* A**64**, 112–122. 10.1107/S0108767307043930 18156677

[bb11] Wagner, H. J. (1962). *Arzneim.-Forsch.* **12**, 1065–1070. 13998243

